# Neglected Anterior Dislocation of the Elbow Joint: Case Report of a Rare Injury

**DOI:** 10.5704/MOJ.1807.016

**Published:** 2018-07

**Authors:** SK Nema, G Behera, M Poduval

**Affiliations:** Department of Orthopaedics, Jawaharlal Institute of Postgraduate Medical Education and Research; ^*^Department of Orthopaedics, SME Tata Consultancy Services, Mumbai, India

**Keywords:** elbow joint, neglected dislocation, triceps, olecranon, humerus

## Abstract

We present an unusual case of five months old neglected anterior dislocation of the right elbow joint in a 19-year old man. The patient had been initially treated by a traditional bone setter, but the elbow remained unreduced. He presented to us with pain, deformity and limited range of motion of his right elbow joint. Radiographs revealed an unreduced anterior dislocation of the right elbow joint. We describe the problems encountered during open reduction and rehabilitation and result one year after the operation with the patient having a stable elbow and a functional range of motion.

## Introduction

The elbow is the second most common joint that sustains dislocation in adults, and the treatment is simple. Old unreduced posterior dislocation of the elbow joint is common in developing countries. The reasons ascribed are the lack of awareness, inadequate access to health care facilities and easy access to traditional bone setters. Acute anterior dislocation of the elbow joint associated with fracture of olecranon have been reported in adults and children^1^. Acute uncomplicated anterior dislocation of the elbow joint is rare compared to its posterior counterpart and good treatment outcomes have been reported^2-4^. Old unreduced anterior dislocation of the elbow joint has not been reported. Here we discuss the likely mechanism, clinical features, disability at presentation, challenges anticipated, problems encountered and result after open reduction in a five-month old unreduced anterior dislocation of the elbow joint in a 19-year old male patient.

## Case Report

A 19-year old male presented to us in the outpatient department with complaints pain on lifting weight with the right arm, deformity and limited range of motion of the right elbow for five months. The patient had fallen down and sustained the injury to his right elbow while hanging from the rootlets of a Banyan tree, following which, he had pain, swelling, and deformity of the right elbow. He had sought treatment from a local bone setter for four weeks following which pain and swelling decreased, but the deformity and elbow stiffness had persisted, for which he attended our hospital.

On examination, the Beighton hyperlaxity score of the patient was 5/9. There was flexion deformity of the elbow joint and wasting of muscles of the arm and forearm. The olecranon process was displaced from the olecranon fossa of the right humerus and an abnormal bone mass was palpable on the anterior aspect of the distal humerus. There was a flexion deformity of 40 degrees of the elbow joint with further flexion of 70 degrees. Pronation and supination were normal. There was a valgus laxity of the right elbow joint. The differential diagnoses were neglected dislocation of the elbow joint (posterior/anterior) and mal-united supracondylar fracture.

**Fig. 1:**
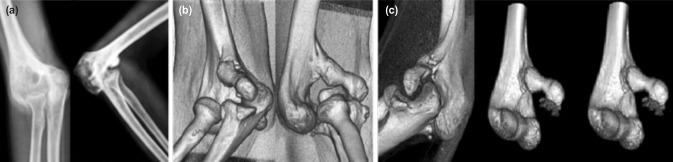
(a, b) AP and lateral radiograph and three dimensional reconstruction (3D) CT scan showing anterior dislocation of the elbow joint. (c) 3D CT reconstruction with subtraction of radius and ulna anteroposterior and lateral views demonstrating anatomy of the distal humerus.

Antero-posterior and lateral radiographs of right elbow demonstrated an anterior dislocation of the elbow joint with an anterior bone mass at the distal humerus. The bony anatomy of the elbow appeared unclear on radiography, and a Computed Tomogram (CT) with 3D reconstruction (Fig. 1) confirmed an anterior dislocation of the right elbow joint with a bony projection from the anterior border of the distal humerus. We hypothesised that because of hyperlaxity the patient had sustained anterior dislocation of the elbow joint without associated fracture. Massage and attempts to reduce the elbow joint by the bone setter had led to the formation of a heterotopic bone mass on the volar aspect of the humerus.

We performed an open reduction of the elbow by combined medial and lateral approach based on findings of the CT scan. We were successful in excising the bone mass but failed to reduce the elbow joint. There was some early degeneration of the articular cartilage of the distal humerus and olecranon. It was impossible to reduce the olecranon posteriorly. We extended the approach through the subcutaneous plane to the posterior aspect and performed an olecranon osteotomy. The humerus was reduced into the osteotomy, and it was fixed with tension-band wiring. Indomethacin was started at 25mg eight hourly after surgery for three weeks after the operation. We did not immobilise the elbow and started active assisted mobilisation of the elbow joint after surgery as tolerated by the patient. The patient was discharged after wound inspection on the 5th post-operative day and advised to attend the rehabilitation department for physiotherapy for six weeks.

**Fig. 2:**
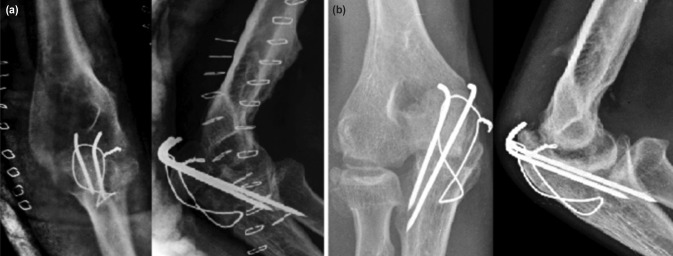
Radiographs (a) After operation. (b) At the end of one year showing arthritic changes.

At review one year postoperative he had a painless range of motion of 30 degrees to 120 degrees at the elbow joint. He has excellent pronation and supination and could perform light activities. The olecranon osteotomy healed well (Fig. 2) though there was a reduction in the joint space of the elbow.

## Discussion

Acute anterior dislocations of the elbow joint have been described in the literature^2-4^. Venkatram *et al* have described the mechanism of anterior dislocation in a 1-year old child. They hypothesised that a sudden pull on the forearm in an attempt to stop the child from running and falling down on the floor might cause an anterior dislocation at the elbow join^t^2. We believe that a mechanism similar to the one described by them operated in our case. An association of fractures of the olecranon, medial epicondyle, lateral epicondyle and pulled elbow has been reported with anterior dislocation of the elbow joint^1-5^.

Clinical features described for an acute anterior dislocation of the elbow joint are a flexed attitude, pain, swelling, deformity and painful restriction of range of motion. Anatomically there is disruption of the bony relationship between the olecranon, the lateral and the medial epicondyles^5^. The case described here presented with similar clinical and anatomical findings. The delay in seeking treatment and presence of bony mass anteriorly at the distal humerus posed a challenge in arriving at the diagnosis of elbow dislocation. Good outcome has been reported in neglected posterior elbow joint dislocations after open reduction utilising the posterior approach with or without V-Y plasty. We had several challenges in deciding the surgical approach, extent of soft tissue release and duration of postoperative immobilisation in our case. We planned open reduction of the elbow by utilising combined medial and lateral approaches because of the position of olecranon and radial head in an antero-medial location. A posterior approach in itself would have severely hampered the access anteriorly. We believe that antero-medial displacement of olecranon and radial head in relation to the distal humerus led to stretching of the triceps. A triceps split or a V-Y plasty of the triceps would have been probably insufficient and challenging to bring the trochlea into the olecranon. However, the inability to reduce the humero-ulnar articulation despite complete release made us apply the unconventional step of an olecranon osteotomy through the existing medial limb of the combined medial and lateral approach.

To summarise, anterior dislocation of the elbow joint is infrequently reported. Treatment of unreduced anterior dislocation of the elbow joint can be a challenging problem. A good outcome can be expected in adequately treated cases.

## Conflict of Interest

The authors declare no conflicts of interest.
